# Rates of refusal of clinical autopsies among HIV-positive decedents and an overview of autopsies in Uganda

**DOI:** 10.12688/wellcomeopenres.17316.2

**Published:** 2022-02-01

**Authors:** Olivie C. Namuju, Richard Kwizera, Robert Lukande, Katelyn A. Pastick, Jonee M. Taylor, Melanie R. Nicol, David R. Boulware, David B. Meya

**Affiliations:** 1 1. Infectious Diseases Institute, College of Health Sciences, Makerere University, Kampala, Uganda; 2 2. Department of Pathology, Makerere University, Kampala, Uganda; 3 3. Division of Infectious Diseases and International Medicine, Department of Medicine, University of Minnesota, Minneapolis, Minnesota, USA; 4 4. Department of Experimental and Clinical Pharmacology, College of Pharmacy, University of Minnesota, Minneapolis, Minnesota, USA; 5 5. Department of Medicine, School of Medicine, College of Health Sciences, Makerere University, Kampala, Uganda

**Keywords:** Autopsy, postmortem changes, autopsy refusal, mortality, HIV Seropositivity, Uganda, Africa South of the Sahara, Investigative Techniques

## Abstract

**Background:** Human immunodeficiency virus (HIV)-related mortality remains high in sub-Saharan Africa. Clinical autopsies can provide invaluable information to help ascertain the cause of death. We aimed to determine the rate and reasons for autopsy refusal amongst families of HIV-positive decedents in Uganda.

**Methods:** We consented the next-of-kin for post-mortem examinations among Ugandan decedents with HIV from 2017-2020 at Kiruddu National Referral Hospital. For those who refused autopsies, reasons were recorded.

**Results:** In this analysis, 165 decedents with HIV were included from three selected wards at Kiruddu National Referral Hospital.  Autopsy was not performed in 45% of the deceased patients; the rate of autopsy refusal was 36%. The most common reasons for autopsy refusal were time constraints (30%), family satisfaction with clinical diagnosis (15%), fear of disfigurement of the remains (15%), and lack of perceived benefit (15%). By seeking consent from multiple family members and clearly explaining to them the purpose of performing the autopsy, we found a reduction in the rate of autopsy refusal among relatives of the deceased patients at this hospital compared to previous studies at the same site (36% vs. 60%).

**Conclusions: **We found lower rates of autopsy refusal compared to previous studies at the same site. This underscores the importance of clearly explaining the purpose of autopsies as they increase active sensitization about their relevance and dispel myths related to autopsies among the general population. Good, culturally sensitive, and timely explanations to the family of the benefits of autopsy increase the rate of obtaining permission. Building capacity for performing autopsies by training more pathologists and increasing laboratory resources to decrease the turn-around-time for autopsy reports and extending these services to peripheral health facilities could improve autopsy acceptance rates.

## Introduction

In sub-Saharan Africa (SSA), the burden of the human immunodeficiency virus (HIV) and HIV-related opportunistic infections remains high ^1, 2^. In 2019, approximately 690,000 people died from AIDS-related illnesses worldwide, including 440,000 in SSA and 23,000 in Uganda ^3^, despite the increased availability of antiretroviral therapy (ART) and the recommendation to treat all HIV-positive individuals regardless of their CD4 ^+^ T-cell counts ^4^.

Clinical autopsies consist of a thorough examination of the decedent to determine the cause of death and evaluate the mechanism of death for research, epidemiological, and/or legal purposes. Clinical autopsies give insight into the pathological processes and can determine what factors contributed to a patient's death. Verbal autopsies are commonly used in Uganda ^5^, though they are often incomplete and inaccurate, and may only yield a presumed or probable cause of death ^6^. Clinical autopsies are important since the clinical determination of death may not be the actual cause of death and there can be a discrepancy between the presumed clinical cause of death and the pathological cause of death. Only a clinical autopsy is specific enough to unmask those differences. Among individuals with HIV in SSA, there is a high discrepancy between clinical and autopsy diagnoses, and scientists emphasize the need for reliable information on causes of death in order to improve HIV patient care, guide further research, and inform health policy ^7^. However, despite the vital role played by clinical autopsies in the development of science and practice of medicine, autopsy rates have been declining globally in recent decades ^8^. The decline is attributed to various complex reasons such as costs, advances in diagnostic methods, shortage of trained pathologists and interested pathologists in autopsies, among others ^9– 12^.

 In Uganda, additional factors play a role in decline of number of autopsies performed, such as lack of trained personnel, a limited number of pathology services, administrative challenges in requesting autopsy, fear of mutilation, concerns about delaying the funeral, and insufficient family financial resources ^7, 8^. Previous autopsy studies done in Uganda showed a 38% acceptance rate under study/research conditions and 5% under routine inpatient conditions ^13^. The most frequent (59%) reason for refusing the autopsy was ‘to avoid delays for the burial’.

We, therefore, conducted a study to determine the current rate of and reasons for autopsy refusal under routine inpatient conditions among decedents with HIV. We also discuss an overview on autopsies in Uganda based on our own experiences, highlighting the misconceptions, potential benefits, and challenges associated with performing autopsies in Uganda. 

## Methods

### Study design and procedures

This was a sub-study nested under a prospective observational cohort study conducted in Uganda among inpatients on the infectious diseases, pulmonary, and emergency wards at Kiruddu National Referral Hospital from February 2017 to August 2020 ^14^. The study included all HIV patients who died during this time frame on these three wards. We consecutively selected all eligible participants using purposive sampling during the study period without calculating sample size. Every morning as part of this study, we checked the daily ward report book to look out for any deaths of HIV infected patients. This was repeated several times during the course of the day. For any identified case, we picked the patient file from the records department using the patient’s name and hospital number, then checked for the contacts of the deceased family or next of kin to call them and find out whether they were still in the hospital premises. Those who were still in the hospital premises were approached to seek for informed consent. Routinely, consent is always obtained from the next-of-kin that are available on the wards after the death has occurred (consent to the study and consent to autopsy are the same thing in this context). However, the next of kin who had already left the hospital premises were called on phone to seek for autopsy verbal consent and also request them to come back and give written consent. Before consenting for study participation, we offered bereavement counseling. All of the autopsies and transport of the decedents were paid for by the study. Patients with trauma that would preclude tissue collection or impair diagnostic analysis were excluded. Patients’ demographics, such as sex, age, and HIV/ART history, were recorded from hospital charts. Dates of admission and death were collected by hospital records. Next of kin were given verbal feedback about the outcome of the autopsy done.

For those who refused to have an autopsy performed for their deceased relative, the reasons for refusing were recorded. This was done as a non-structured interview by one of the nurses (OCN) in a private room. It involved only one open ended question, i.e, "why wouldn’t you want an autopsy performed on your deceased relative?". This took about 5–10 minutes, and their responses were written on the screening log without audio/video recording.

To reduce sampling bias, we used the three wards that accommodate more than 95% of all HIV patients in this hospital. The records staff doublechecked all entries by medical staff in the daily ward report book. We carried out the study at a national referral hospital which gets patients from all over the country and could be representative of the whole country.

Outside the study settings, in routine practice, the autopsy procedure is as follows:

- Upon a death occurring in the hospital ward, the treating physician/clinician requests for an autopsy (by filling in the autopsy request form). The request is based on several reasons that include lack of a clinical diagnosis, death within 24 hours of admission on the ward etc.
- The next-of-kin of the deceased are then approached for consenting after receiving an explanation for the need of an autopsy
- Upon signing the consent, the request and consent form are forwarded to the mortuary to be received by the pathology team
- The pathology team (Pathologist and mortuary technicians) prepare for and conduct the autopsy.

### Ethical considerations

All caregivers/next-of-kin of the deceased patients provided written informed consent (the consent form can be found as *Extended data* ^15^). Ethical approval occurred from the Uganda National Council of Science and Technology (HS24ES), and Mulago Hospital Research and Ethics Committee (MHREC 1023).

### Statistical analysis

Investigators had full access to the database population used to create the study population to extract the patients’ data. Data cleaning was mainly done on the responses for “reasons for autopsy refusal” by modifying responses that mean the same to look alike. Data were then analyzed using STATA version 14 (STATA, College Station, Texas). The rate of autopsy refusal and distribution of baseline demographic characteristics were reported as proportions. Frequencies and percentages were reported for each baseline characteristic when considered categorical, and medians (interquartile range) for continuous variables. 

## Results

### Patients’ characteristics

This analysis included 165 deceased HIV-positive patients who died while on the emergency, pulmonary, and infectious disease wards at Kirrudu National Referral Hospital from February 2017 to August 2020. Of those deceased patients with available demographic data (n=119), 55% (65/119) were male with an overall median age of 37 years (n=118; IQR= 30 to 43), and 28% (43/152) were ART naïve ( Table 1) ^16^.

**Table 1.** Characteristics of the study population. HIV=human immunodeficiency virus; IQR=interquartile range.

Characteristics	N with data	N (%) or Median (IQR)
Men	119	65 (54.6%)
Age, years	118	37 (30, 43)
Receiving HIV therapy	152	109 (71.7%)
Length of Hospitalization, days	118	6 (2, 13)
Autopsy performed	165	90 (54.6%)
Time to autopsy, days	90	
Performed same day of death		69 (76,7%)
Performed one day following death		21 (23,3%)

The median length of hospitalization for all deceased patients was 6 days (n=118; IQR= 2 to 13). Of the 165 deceased HIV-positive patients, 55% (n=90) of their relatives consented to autopsy procedures; 45% (n=75) of autopsies were not performed for refusal of autopsy amongst other various reasons ( Table 2). For those who had an autopsy performed, the days to an autopsy from time of death ranged from zero (0) to one day, with the majority (76.7%) performed on the same day as the patient death ( Table 1).

**Table 2.** Reasons for not performing autopsy among human immunodeficiency virus (HIV)-positive persons and rate of autopsy refusal.

Reasons for not performing autopsy * (N=75)	n (%)
Family refused autopsy	27 (36%)
Death occurred at night, family departed before approached for consent, or body already embalmed by the morgue attendants	24 (32%)
Pathologist unavailable	15 (20%)
No family to consent	6 (8%)
No assistant in morgue	3 (4%)
Research-specific exclusion criteria	3 (4%)
Language barrier with the available family members	1 (1.3%)
Reasons for refusal of autopsy (n=27)	n (%)
Time constraint, inability to wait for procedure or location of burial was distant	8 (29.6%)
Family satisfied with the clinical diagnosis	4 (14.8%)
Fear of disfigurement of remains	4 (14.8%)
Family saw no direct benefit in autopsy	4 (14.8%)
Religious beliefs (e.g., Muslims do not accept their deceased bodies to be cut)	2 (7.4%)
Previous bad experience of family members with autopsy or procedures	2 (7.4%)
Lacked clear explanation regarding clinical diagnosis	1 (3.7%)
Death was expected	1 (3.7%)
No reason given	1 (3.7%)

Data presented are numbers. *For four of the patients, two reasons were given for each.

### Rate of autopsy refusal and reasons for not performing an autopsy

Of the 165 deceased HIV patients, 75 (45.4%) did not have an autopsy performed for various reasons ( Table 2). The refusal of autopsy by family members was the most common reason for not performing autopsies (36%; 27/75), followed by the deceased person having been removed from the mortuary before next-of-kin was approached for informed consent (24/75) and the absence of a pathologist (15/75). For four of the deceased patients, two reasons were given for refusal for each. The most common reasons given for autopsy refusal were; time constraints and distant location of internment; family satisfaction with the clinical diagnosis; fear of body mutilation; and many families did not perceive any benefit in having an autopsy since it would not bring back the deceased.

## Discussion

### Overview of clinical autopsies in Uganda

Clinical trials currently provide a platform for improving outcomes for individuals with HIV in Uganda ^17, 18^. Therefore, combining post-mortems with clinical studies is one way to increase uptake of autopsies. In this study, we observed a lower rate of autopsy refusal (36%) under inpatient conditions compared to the previous studies in the same setting which had a higher rate (60%) during the period May–September 2009 ^13^. We attribute this reduction to seeking consent from responsible family members/elders other than from first-line caretakers only, a more thorough explanation of the purpose of autopsies, and reassurance to the family that no cost would be incurred for the autopsy or embalming.

For those who refused an autopsy for their deceased relative, in this study, we found that the most common reasons were due to time constraints and distant location of internment, in addition to families’ satisfaction with the clinical diagnosis of HIV and HIV-related opportunistic infections, and fear of body mutilation. Failure of families to perceive any benefit of the autopsy could partly be seen as caregivers of the deceased expressing “care-fatigue,” especially when it was among relatives who had been ill for a long time. However, we posit that active sensitization about the relevance of autopsies in the general population, emphasizing the point that the clinical diagnosis may not necessarily be the cause of death, will increase the likelihood of consent for autopsies. Healthcare workers need to clearly explain the purpose of performing autopsies. In our setting, next of kin were given verbal feedback after the autopsy was done. We believe that this provided closure for most of them especially those whose patients died after a few days of hospitalisation. However, this cannot be confirmed since there was no questionnaire administered after the autopsy had been done, to assess the usefulness of the information given. Worth still, for future studies, we plan to incorporate feedback from the family members, and although, this was outside the scope of the current study. The concern of body mutilation during the autopsy with some parts of the deceased being retained by the pathologist was raised by several relatives. This reason was more frequent among Muslim families. The concern for body mutilation was also noted among healthcare workers, which may influence their attitude towards requesting autopsies when patients they are treating die of unknown causes. The issue of pathologists retaining body organs is a myth. People think that entire organs are retained yet only very tiny tissues from each organ are taken off and the rest of the organs are put back.

In Uganda today, verbal autopsies are more commonly used in peripheral health facilities based on clinical and/or confirmed laboratory diagnosis ^5^. However, verbal autopsies have limitations as they are not always reliable/accurate, may be incomplete, cannot be replicated, and are often problematic with diseases that have less specific symptoms, hence only a presumed or probable cause of death may be given ^6^. Despite these limitations, clinical autopsies are infrequently performed at regional and national referral hospitals in Uganda. The majority of autopsies are performed on request for either research purposes or forensic medicine. Yet, they would be useful in targeted patients’ populations given the uncertainty of infectious causes of death and unknown pathologies.

Similarly, the high cost of the autopsy procedure (approximately 162 USD), is out of reach by most families who have relatives admitted in public health facilities especially given the out-of-pocket expenses incurred for treatment of the decedent. This prohibitive cost contributes to the increased rates of decline for autopsies. Having the cost of the procedure subsidized as part of healthcare costs incurred by public health care facilities could help to solve this. Assurance should be given to families that no extra charges will be encountered to perform the treatment and autopsy understudy settings. Similarly, expanding the capacity of health facilities to perform autopsies in terms of having equipped morgues and more trained personnel to perform autopsies while extending these services to peripheral health centers could potentially improve autopsy acceptance rates. However, an unknown, but presumably significant, number of patients die in their communities at home without the relatives of the deceased seeking to have an autopsy or embalming services from health facilities. This practice has not only occasionally been the focal point of infectious disease epidemics, but this lack of documentation of deaths also underestimates the burden of disease in the country.

As observed in this study, there is a shortage of logistical support in terms of basic medical and laboratory supplies and personal protective gear to favor autopsy procedures for all hospitalized who die. The small number of trained pathologists in the country is a major hindrance to autopsies since the pathologists tend to be overstretched, especially when forensic autopsies are ongoing. In most cases, morgue attendants are quick to embalm the deceased shortly after death, either due to the absence of a healthcare worker to order an autopsy or due to pressure from the family of the deceased, who typically want to leave the hospital as soon as possible to arrange the funeral. This practice does not allow sufficient time for the doctors to discuss the reasons and obtain consent for autopsies. Finally, dilapidated morgues at most public health care facilities are a deterrent to encourage autopsies at these facilities.

### Misconceptions about clinical autopsies in Uganda

A number of misconceptions surround autopsies in Uganda. According to our respondents or family members, most people believe that when autopsies are performed, all internal organs are removed and replaced with cotton wool. In some communities, autopsies are completely unacceptable culturally or are perceived as taboo. Some religions, including Islam, consider autopsies as an unacceptable practice for Muslims because they believe the dead have to return whole just as they were born. There is also a myth about the deceased coming back to torment the family members because of the procedures performed on the deceased relatives’ body. 

### Potential benefits of clinical autopsies

Autopsies can be beneficial in understanding the primary cause of death, which may be different from the clinical diagnosis. Establishing and understanding the primary cause of death, in turn, helps to improve the care of patients still living with the disease. Autopsies aid improvement in diagnosis, a better understanding of disease progression, and the development of more targeted therapies, which reduce mortality and save lives in the future. Of importance in forensic medicine, autopsies provide evidence that helps to apprehend criminals and/or by establishing the true cause of death, puts suspicions to rest. Autopsy reports are also important in enabling the next-of-kin to obtain official certification of death, which may be required as part of the administrative processes of the estate of the deceased. Autopsy reports are used as part of hospital audits to identify areas of improvement and the gaps that need to be bridged.

### Challenges in consenting and performing autopsies in Uganda

It is often emotionally difficult for the nurse or doctor to talk to grieving family members, especially when a death has just occurred. Despite the lengthy consenting process involved in this study, we endeavored to provide bereavement counseling first to comfort the family before discussing the importance of performing an autopsy for their deceased relative. We learned early on that understanding family dynamics is important, given the communal setting in Uganda. In order for the consenting process to be successful, we sought consent from the first line caregivers but also ensured we identified the family decision-makers and involved them in these discussions for the autopsies.

Additional challenges we noted were the prolonged waiting time for families at the morgue to receive the decedent and system constraints. We found the wait time to be typically 4 hours, which may be inexplicably long, creating anxiety and consternation. Kiruddu/ Mulago National Referral Hospital has about 14 pathologists only, which means the few pathologists have a heavy workload, which contributes to delayed or missing final autopsy reports. Nationally, the number of Anatomical Pathologists in the country is low and indeed those that are actively involved in autopsy work is low. Autopsy work in the Regional Referral and District Hospitals is handled by Medical Officers (MOs) who are not Specialist Pathologists. The Anatomical Pathologists are at the National Referral Hospital and in the Medical School. Lastly, the shortage of instruments and personal protective equipment can expose staff to occupational health hazards and are additional challenges in conducting autopsies in Uganda.

### Limitations to the study

The main limitation to the study is that it was conducted at only one National Referral Hospital among HIV infected patients only, but we believe the challenges discussed here apply to the general population in the context of obtaining autopsies in most resource limited settings. The fact that study staff checked inpatient registers for deaths, actively contacted next-of-kin, offered bereavement counseling, and communicated that no costs would be incurred, all could have positively impacted or overestimated the rate of autopsy acceptance observed. Some patients included had incomplete data sets.

## Conclusion

Clinical autopsies remain relevant procedures to determine the cause of death. In the current study, we observed a lower autopsy refusal rate under normal hospital conditions among HIV-positive patients in Uganda compared to previously reported rates in the same setting. By seeking consent from more family members and clearly explaining to the families the purpose of performing autopsies, we reduced the refusal rate for autopsies among relatives of deceased patients at this hospital. Healthcare workers need to clearly explain the purpose of performing autopsies as they increase active sensitization about the relevance of autopsies and dispel misconceptions related to autopsies among the general population. Building capacity for performing autopsies by training more pathologists and laboratory resources to decrease the turn-around-time for autopsy reports and extending these services to peripheral health facilities could improve autopsy acceptance rates in Uganda.

## Data availability

### Underlying data

Figshare: Autopsy Data 2020 CLEANED (2).xlsx. https://doi.org/10.6084/m9.figshare.16929499.v1 ^16^.

### Extended data

Figshare: Approved English consent 2020.pdf. https://doi.org/10.6084/m9.figshare.16904731.v1 ^15^.

Data are available under the terms of the Creative Commons Zero "No rights reserved" data waiver (CC0 1.0 Public domain dedication).
