# Supplementary material for: Rates of refusal of clinical autopsies among HIV-positive decedents and an overview of autopsies in Uganda
Source: Wellcome Open Res. 2022 Feb 1;6:302. Originally published 2021 Nov 9. [Version 2] doi: 10.12688/wellcomeopenres.17316.2 (PMC9086520; doi:10.12688/wellcomeopenres.17316.2)
Supplement: Peer review history

# Peer review: Reviewer response for version 2

**DOI:** 10.21956/wellcomeopenres.19472.r50121

**Published:** 2022-06-06

**Authors:** Dr Corinne L. Fligner, MD

**Affiliations:** 1 Professor Emerita, Department of Laboratory Medicine and Pathology, University of Washington, Seattle, WA, USA

This is a 3-year study (2017-2020) focused on identifying the rate of and reasons for the refusal of autopsy by families of hospitalized HIV-positive decedents in Uganda. This research group embedded the study in a prospective observational cohort study of HIV inpatients who died in a National Referral Hospital in Uganda. A similar study of autopsy rate and reasons for autopsy refusal was done at another National Referral Hospital in Uganda for five months in 2009. In contrast, the present study included more patients over a longer period of time and was done as part of another clinical research study. In addition, there was modification of the procedures for obtaining consent to include culturally sensitive and clear discussion of autopsy with responsible family members, as well as procedural changes to emphasize timely performance of autopsy and communication of the results to family members. The authors identified a reduction in the rate of autopsy refusal from the previous study from 62% to 36%. Thus, the autopsy acceptance rate by families in this study who were asked for autopsy consent was 64%, which is quite remarkable. 

 This study was well-designed and implemented; data collection, presentation, and analysis were clear, and the evaluation of the results and conclusions are well-founded.  Importantly, the methods are clearly delineated so that they can be used as a basis for similar studies by these or other investigators. There are very few quality studies describing the reasons for autopsy acceptance or refusal by families in low resource countries. The value of clinical autopsies in determining the accurate cause of death in this and other studies has been well-established. Continued assessment and modification of autopsy consenting practices as described in this paper are important to facilitating research utilizing autopsy information to improve patient care and public health.

Is the work clearly and accurately presented and does it cite the current literature?

Yes

If applicable, is the statistical analysis and its interpretation appropriate?

Yes

Are all the source data underlying the results available to ensure full reproducibility?

Yes

Is the study design appropriate and is the work technically sound?

Yes

Are the conclusions drawn adequately supported by the results?

Yes

Are sufficient details of methods and analysis provided to allow replication by others?

Yes

Reviewer Expertise:

Adult and perinatal-fetal autopsy pathology; forensic pathology

I confirm that I have read this submission and believe that I have an appropriate level of expertise to confirm that it is of an acceptable scientific standard.

# Peer review: Reviewer response for version 2

**DOI:** 10.21956/wellcomeopenres.19472.r50118

**Published:** 2022-05-16

**Authors:** Omar K. Siddiqi

**Affiliations:** 1 Global Neurology Program, Department of Neurology, Beth Israel Deaconess Medical Center, Boston, MA, USA

The authors have completed an assessment, nested in a larger study of deceased HIV patients, of factors related to autopsy refusal among HIV-infected patients in Uganda. This is an important topic of inquiry because, as the authors have stated, the rate of complete diagnostic autopsies (CDA) is declining worldwide. In addition, CDA provides a level of granularity of findings that is absent in verbal autopsies. This is particularly useful in patients with advanced HIV infection who can have multiple complex factors that contribute to death. The study findings are important and the authors have responded appropriately to the response of the prior reviewer.

 I have the following suggestions for improvement:

1. On page 3, paragraph 2 of the Introduction, the authors state that one of the reasons why the rates of CDA are declining worldwide is due in part to the lack of “interested pathologists in autopsies”. I am unclear on how, outside of a research study, pathologist interest in autopsies relates to volume. Pathologists do not request autopsies as this is normally done by treating providers, family, or police. Please clarify or reword to help explain what is meant.
2. On page 4, paragraph 3 of the Results, “the absence of a pathologist” was given as a reason for not performing autopsies. Organ harvesting is commonly done by technicians and pathologists can cut tissue and review slides later. Does a pathologist need to be present for organ collection at this facility?
3. On page 5, paragraph 2 of the Discussion, the word “internment” is awkward and does not make sense in the context of the sentence. Please reword.
4. On page 5, paragraph 4 of the Discussion, the authors reference a “high cost” for an autopsy. Why does the autopsy cost anything? Often, this is a service provided by the hospital. What do these costs cover?
5. On page 6, paragraph 1 of the Discussion, the authors discuss the issue of community deaths that are known to frequently occur in sub-Saharan Africa. Tertiary care centers in some countries record those brought in deceased. Does this exist in the relevant facilities in Uganda? If so, please mention it in more detail as this can be more reflective of overall causes of mortality than just facility deaths.
6. On page 6, paragraph 2 of the Discussion, the authors reference “forensic autopsies”. It would be useful to know how many of these are done and who pays for the costs.
7. On page 6, paragraph 6 of the Discussion, the authors state that nationally the number of anatomical pathologists is “low”. If the exact or estimated number can be provided, this would be useful as a comparison to neighboring countries.
8. I would encourage the authors to mention minimally invasive tissue sampling (MITS), also known as minimally invasive autopsy (MIA), in the Discussion as a feasible alternative to CDA that has been shown to have comparable validity to CDA, particularly related to infectious causes of mortality, and has successfully been implemented in multiple SSA countries. It provides multi-site core biopsy samples from relevant tissue in under one hour without desecration of the body. This could be a solution to some of the barriers mentioned in this study to adequately determine the cause of death ^1^^,^] ^2^^,^^3^.

Is the work clearly and accurately presented and does it cite the current literature?

Yes

If applicable, is the statistical analysis and its interpretation appropriate?

Yes

Are all the source data underlying the results available to ensure full reproducibility?

Yes

Is the study design appropriate and is the work technically sound?

Yes

Are the conclusions drawn adequately supported by the results?

Yes

Are sufficient details of methods and analysis provided to allow replication by others?

Yes

Reviewer Expertise:

Central nervous system infections. I have also been engaged in autopsy work in a similar population in Zambia.

I confirm that I have read this submission and believe that I have an appropriate level of expertise to confirm that it is of an acceptable scientific standard.

## References

[1] : Non-invasive or minimally invasive autopsy compared to conventional autopsy of suspected natural deaths in adults: a systematic review. *Eur Radiol* .2016;26(4) : 10.1007/s00330-015-3908-8 1159-79 10.1007/s00330-015-3908-8 26210206PMC4778156

[2] : Infectious cause of death determination using minimally invasive autopsies in developing countries. *Diagn Microbiol Infect Dis* .2016;84(1) : 10.1016/j.diagmicrobio.2015.10.002 80-86 10.1016/j.diagmicrobio.2015.10.002 26508103

[3] : Validity of a minimally invasive autopsy for cause of death determination in maternal deaths in Mozambique: An observational study. *PLoS Med* .2017;14(11) : 10.1371/journal.pmed.1002431 e1002431 10.1371/journal.pmed.1002431 29117196PMC5695595

# Peer review: Reviewer response for version 2

**DOI:** 10.21956/wellcomeopenres.19472.r50120

**Published:** 2022-05-09

**Authors:** Edwin Oloo Walong

**Affiliations:** 1 Anatomic Pathology Unit, Department of Human Pathology, School of Medicine, University of Nairobi, Nairobi, Kenya

I would like to congratulate the study team for submitting this manuscript. This article explores a common problem in African autopsy practise and presents hypothesis that should be evaluated further by a formal social science research team. The data presented shows reasons for refusal, while table 1 presents a summary of the characteristics of the study population. While not much data is presented, this information is sufficient to support the conclusions made, and raises questions necessary for further study.

Is the work clearly and accurately presented and does it cite the current literature?

Yes

If applicable, is the statistical analysis and its interpretation appropriate?

Yes

Are all the source data underlying the results available to ensure full reproducibility?

Yes

Is the study design appropriate and is the work technically sound?

Yes

Are the conclusions drawn adequately supported by the results?

Yes

Are sufficient details of methods and analysis provided to allow replication by others?

Yes

Reviewer Expertise:

Autopsy Pathology, Infectious Disease Surveillance

I confirm that I have read this submission and believe that I have an appropriate level of expertise to confirm that it is of an acceptable scientific standard.

# Peer review: Reviewer response for version 2

**DOI:** 10.21956/wellcomeopenres.19472.r48434

**Published:** 2022-02-02

**Authors:** Ross M. Boyce

**Affiliations:** 1 Department of Epidemiology, Gillings School of Global Public Health, University of North Carolina at Chapel Hill, Chapel Hill, NC, USA; 2 Division of Infectious Diseases, University of North Carolina School of Medicine, Chapel Hill, NC, USA; 3 Department of Community Health, Faculty of Medicine, Mbarara University of Science and Technology, Mbarara, Uganda

The authors have addressed my comments.

Is the work clearly and accurately presented and does it cite the current literature?

Yes

If applicable, is the statistical analysis and its interpretation appropriate?

Yes

Are all the source data underlying the results available to ensure full reproducibility?

Yes

Is the study design appropriate and is the work technically sound?

Yes

Are the conclusions drawn adequately supported by the results?

Partly

Are sufficient details of methods and analysis provided to allow replication by others?

Yes

Reviewer Expertise:

Malaria, Vector-borne diseases, Infectious diseases epidemiology, surveillance

I confirm that I have read this submission and believe that I have an appropriate level of expertise to confirm that it is of an acceptable scientific standard.

# Peer review: Reviewer response for version 1

**DOI:** 10.21956/wellcomeopenres.19144.r47002

**Published:** 2021-11-16

**Authors:** Ross M. Boyce

**Affiliations:** 1 Department of Epidemiology, Gillings School of Global Public Health, University of North Carolina at Chapel Hill, Chapel Hill, NC, USA; 2 Division of Infectious Diseases, University of North Carolina School of Medicine, Chapel Hill, NC, USA; 3 Department of Community Health, Faculty of Medicine, Mbarara University of Science and Technology, Mbarara, Uganda

The submitted manuscript reports the rate of clinical autopsies performed among HIV-positive decedants at the national referral hospital in Uganda, while also exploring reasons that autopsies were not performed, including both administrative/logistical issues and the stated rationale of next-of-kin. Understanding cause of death is an important, albeit somewhat neglected, topic both for routine disease surveillance and clinical/epidemiological research and the authors should be commended for nesting this work within the parent study. The manuscript is well organized and written. The results are straightforward and presented in a clear manner. The conclusions are generally appropriate to the results presented, although I believe the generalizability of the findings may be more limited than the authors suggest, as noted in my comments below. 

**Major Comments:**

1. The study site, located in close proximity to the capital city and co-located with the most prestigious university/medical school, is the most capable and well-resourced public health facility in Uganda. While the authors state that MNRH has "only" 14 pathologists, I suspect that the majority of regional referral centers, district hospitals, and other such facilities that care for inpatients have much fewer, if any, trained pathologists. Thus, the findings are likely to be a best-case estimate of autopsy rates, especially in regard to the issue of overburdened clinical staff, which accounted for ~25% of autopsy non-performance. 
2. Similarly, I suspect that study methodology in which study staff checked inpatient registers for deaths, actively contacted next-of-kin, offered bereavement counseling, and communicated that no costs would be incurred all positively impacted the rate of autopsy acceptance. This is a relatively unavoidable consequence of the study design (and appropriate to ethical requirements), but again, probably overestimates the "real world" (i.e., non-study) rate of autopsy. This should be mentioned in the limitations section.
3.  "We carried out the study at a national referral hospital which gets patients from all over the country and could be representative of the whole country." Without data, this is largely speculation. It is well documented that distance/travel burden substantially impacts the ability to pursue higher-level care. In contrast to the statement, I would suspect that most participants were from the immediate environs with a substantial decrease as one moves further from MNRH (i.e., distance decay). Those who were from a greater distance were likely those with more resources available to make the trip and thus are likely to be different. If data is available on district of residence of participants, I would consider including to support (or refute) this assertion.

**Minor Comments:**

1. Methods
  - Unclear if consent in the sentence. "Those who were still in the hospital premises were approached to seek for informed consent" refers to consent for the study or consent for the autopsy?
  - Would be helpful if there was some description of how the process of autopsies is done in routine practice (i.e., outside of this study).
2. Discussion
  - "This reason was notably greater among Muslim families." The stratified data (by religion) to support this conclusion is not presented.

**Questions / Suggestions:**

1. Any reason to think that issues relating to HIV stigma may have influenced family decision to pursue autopsy?
2. Would be valuable to get feedback on Next-of-Kin / Family experiences with autopsy when performed. In other words, did they find it useful or informative? Did it provide closure? This could be helpful in informing future interventions. 
3. It appears that at least a portion of the study period took place during the COVID-19 pandemic. Was there any difference, statistically or anecdotally in the rates or reason for non-performance during the pandemic potentially as a result of fear or national lock downs?
4. The Discussion section outlines many of the current challenges in achieving a high rate of autopsies, but would benefit from further discussion of potential ways/interventions to improve the rate in routine practice.
5. Are non-physician professionals (i.e., clinical officers) who might be able to be trained to perform clinical autopsies?

Is the work clearly and accurately presented and does it cite the current literature?

Yes

If applicable, is the statistical analysis and its interpretation appropriate?

Yes

Are all the source data underlying the results available to ensure full reproducibility?

Yes

Is the study design appropriate and is the work technically sound?

Yes

Are the conclusions drawn adequately supported by the results?

Partly

Are sufficient details of methods and analysis provided to allow replication by others?

Yes

Reviewer Expertise:

Malaria, Vector-borne diseases, Infectious diseases epidemiology, surveillance

I confirm that I have read this submission and believe that I have an appropriate level of expertise to confirm that it is of an acceptable scientific standard, however I have significant reservations, as outlined above.

# Response

**Published:** 2022-01-10

**Authors:** Olivie Carolyne Namuju

**Affiliations:** Makerere University, Uganda

Following these helpful comments from the reviewer, we made a number of tracked changes in v1 of the text. Nationally, the number of Anatomical Pathologists in the country is low and indeed those that are actively involved in autopsy work is low.  Yes, the autopsy work in the Regional Referral and District Hospitals is handled by Medical Officers (MOs) who are not Specialist Pathologists. The Anatomical Pathologists are at the National Referral Hospital and in the Medical School. The findings are indeed a reflection of what happens at the national referral hospital level. Yes, the study methodology could have overestimated the rate of autopsy observed. This has been added to the limitations. The fact that the hospital admits patients across the entire country and of whom were enrolled into the study, we assumed this gives a general representation of those from a greater distance. Consent is always obtained from the next-of-kin that are available on the wards after the death has occurred. Also, consent to the study and consent to autopsy are the same thing in this context. We have also added a brief description of the autopsy procedure outside the study settings, in routine practice.Analysis was not stratified by religion. This has been modified in the text. People tend to associate stigma more with the living than the dead. Next of kin were given verbal feedback after the autopsy was done. It provided closure for most of them especially those whose patients died after a few days of hospitalisation. Yes, there was an increased challenge during the pandemic time mainly because of the difficulties with getting transportation that made next of kin always to be in a hurry before curfews hours. Increasing public awareness on the importance of autopsies preferably through the media would be a solution. Currently in Uganda, autopsies are conducted by Medical Officers and Pathologists. No other cadre is permitted to do otherwise.
